# A Narrative Review of Clinical and Molecular Criteria for the Selection of Poor Candidates for Optimal Cytoreduction in Epithelial Ovarian Cancer

**DOI:** 10.3390/life15081318

**Published:** 2025-08-20

**Authors:** George Pariza, Carmen Mavrodin, Alina Potorac, Octavian Munteanu, Monica Mihaela Cîrstoiu

**Affiliations:** 1 Faculty of Medicine, “Carol Davila” University of Medicine and Pharmacy, 050474 Bucharest, Romania; george.pariza@umfcd.ro (G.P.); alinapotorac05@gmail.com (A.P.); octav_munteanu@yahoo.com (O.M.); monica.cirstoiu@umfcd.ro (M.M.C.); 2 5th Department of General Surgery, Emergency Hospital Bucharest, 050098 Bucharest, Romania; 3 3th Department of General Surgery, Emergency Hospital Bucharest, 050098 Bucharest, Romania; 4 Department of Obstetrics and Gynecology, Emergency Hospital Bucharest, 050098 Bucharest, Romania

**Keywords:** ovarian cancer, surgery, carcinomatosis, frailty, cytoreduction

## Abstract

**Objective:** The objective of this paper is to define “poor candidates” and to conduct an analysis of preoperative selection criteria, considering factors related to the patient, tumor burden, and histopathological characteristics, in the case of patients with advanced epithelial ovarian cancer (EOC) FIGO III-IV with a low probability of optimal cytoreduction. **Methodology:** The authors of this narrative review conducted an analysis of articles published over a 20-year period (2005–2025), with the following selection criteria for the topics of the papers: advanced epithelial ovarian cancer (FIGOIII-IV), surgical indications in advanced ovarian cancer, poor candidates for surgery, and dependence between surgery and histopathologic and molecular type of EOC. They used using PubMed, Science Direct, and Scopus as databases. The results of the analysis were organized into three large chapters that grouped patient-related factors, tumor burden-specific factors, and histopathological criteria. **Results:** The authors identify a series of criteria with a high risk of unfavorable postoperative evolution, which led to delayed chemotherapy treatment and suboptimal management. These criteria are related to the patient’s field (ECOG > 3, Charlson Comorbidity Index (CCI) > 2, BMI > 25–30, hypoalbuminemia, hypokalemia), imaging or intraoperative factors predictive for residual tumor, and histopathological or genetic factors (presence of BRCA mutation favors optimal cytoreduction even in cases with high tumor burden; in the case of low-grade serous ovarian carcinoma, surgical intervention is recommended even if there are suboptimal resection criteria, accepting resection > 1 cm due to the poor response to specific chemotherapy treatment). **Conclusions:** Considering all these aspects, patient selection for primary debulking surgery (PDS) or NACT (neoadjuvant chemotherapy) and interval debulking surgery (IDS) should be conducted in oncological surgery centers highly specialized in gynecological neoplasms, thus ensuring an optimal therapeutic pathway for patients with EOC.

## 1. Objective

The objective of this paper is to define “poor candidates” and to standardize preoperative selection criteria, considering factors related to the patient, tumor burden, and histopathological characteristics, in the case of patients with advanced epithelial ovarian cancer FIGO III-IV with a low probability of optimal cytoreduction.

## 2. Introduction

Given the nonspecific symptomatology, or even the absence of symptoms in the early stages of ovarian cancer, a significant proportion of patients—over two-thirds of all diagnosed cases [1]—present with advanced forms of the disease.

The current standard treatment for EOC in FIGO stages III and IV consists of surgical management as the initial approach, i.e., primary debulking surgery (PDS) with no macroscopic residual disease followed by chemotherapy, this being the most important prognostic factor, or interval cytoreduction, followed or preceded by platinum-based chemotherapy. Exceptions to this protocol include patients initially deemed unresectable or those in whom optimal cytoreduction cannot be achieved.

There is no consensus on the definition of patients who are not eligible for primary intervention, nor a clear stratification of the frailty of these patients.

Current guidelines do not provide standardized criteria for the selection of patients considered frail and for whom PDS may lead to a prolonged recovery period with delayed initiation of chemotherapy. An incomplete or incorrect assessment of resectability and frailty may be followed by a suboptimal resection with residual disease in a patient with a high frailty index, with the association of the two aspects leading to a decrease in the chances of survival.

Considering that 33–56% of women diagnosed with ovarian cancer do not receive treatment consistent with established guidelines [2,3], and that, statistically, 26% of cases detected in stage III and 57% of cases in stage IV receive only systemic therapy without benefiting from primary or interval cytoreduction [2], while 15% of stage III cases and 48% of stage IV cases receive no treatment at all [4], and given that incomplete initial cytoreduction has a negative impact on survival compared to patients who undergo complete cytoreduction, it is essential that the selection of EOC patients—regarding their candidacy for surgery—be performed in specialized centers with expertise in ovarian cancer management [5].

In this context, the objective of this article is to provide an updated overview of the criteria for selecting patients eligible for primary debulking surgery and to systematize current therapeutic recommendations.

## 3. Methodology

The authors conducted a narrative review and analyzed articles published over a 20-year period (2005–2025), with the following selection criteria for the topics of the papers: advanced epithelial ovarian cancer (FIGOIII-IV), surgical indications in advanced ovarian cancer, poor candidates for surgery, and dependence between surgery and histopathologic and molecular type of EOC. They used PubMed, Science Direct, and Scopus as databases. Only articles in English and published in full text were selected. The results of the analysis were organized into 3 large chapters that grouped patient-related factors, tumor burden-specific factors, and histopathological criteria.

## 4. Defining “Poor Candidates” and Low Probability of Optimal Cytoreduction

Complete resection of all macroscopically visible tumor lesions (R0) represents the most important prognostic factor in advanced ovarian cancer, specifically in FIGO stages III and IV. The current therapeutic standard [5] is R0 resection, including extraperitoneal lesions, followed by chemotherapy (carboplatin/paclitaxel).

Furthermore, the EORTC 55,971 and CHORUS trials have demonstrated that neoadjuvant chemotherapy (NACT) followed by interval debulking surgery (IDS) constitutes a feasible alternative, yielding comparable outcomes in terms of overall survival and progression-free survival. This approach is considered appropriate for patients who are not candidates for initial R0 resection due to the distribution of peritoneal carcinomatosis and/or metastatic lesions, or for patients with significant comorbidities or poor general condition that precludes primary surgical intervention [5,6].

Suboptimal resection is not recommended, as it negatively impacts survival outcomes, and unnecessary surgical procedures should be avoided [6].

Given these therapeutic standards, the selection of patients who are unlikely to benefit from primary debulking surgery (PDS) becomes critically important in order to avoid surgical interventions that do not confer a prognostic advantage over chemotherapy alone and are associated with increased morbidity and mortality, potentially delaying the initiation of oncologic treatment.

Patient evaluation should be based on factors related to overall performance status, tumor stage characteristics, and the likelihood of achieving complete (R0) resection.

### 4.1. Patient-Related Factors—Performance Status

The ESMO-ESGO guidelines recommend that the selection of patients with advanced FIGO stage III and IV ovarian cancer for either primary surgery or chemotherapy followed by interval debulking surgery should be conducted in specialized ovarian cancer centers, considering the complexity of surgical procedures in relation to patient frailty [5].

Moreover, D.J. Shadowitz et al., in a study published in 2016, emphasized that a subset of patients with advanced disease and/or significant comorbidities who received non-surgical management alone demonstrated better survival compared to those who underwent surgical management [4].

The clinical course of patients with ovarian cancer is influenced by multiple patient-specific factors. Those most strongly correlated with increased postoperative morbidity and mortality include ECOG performance status, frailty associated with advanced age, and the presence of comorbidities. All these parameters are considered independent prognostic factors and must be carefully evaluated when establishing the therapeutic strategy [7,8].

Irodi et al. [9], in a prospective study conducted between 1981 and 2015, reported an increase in the age at diagnosis, which was associated with diagnosis at more advanced disease stages and, consequently, higher ECOG (Eastern Cooperative Oncology Group) performance scores at presentation.

Evaluation of oncology patients, regardless of cancer type, undergoing specific therapy, has demonstrated increased utilization of emergency department services, higher rates of readmission within 30 days, and greater mortality among patients with ECOG performance status 3 or 4 compared to those with a performance status of 0 or 1 [10].

In a study published by Inci et al. in 2021 [11], involving 144 patients, it was identified that a frailty score > 0.26 was associated with severe postoperative complications. Furthermore, a frailty index > 0.15, in conjunction with an ECOG performance status > 1, hypoalbuminemia (albumin < 35.5 g/dL), and residual disease > 1 cm, were predictive factors for poorer survival outcomes [11].

Multiple studies have investigated postoperative outcomes in relation to performance status, complexity of associated comorbidities, and the nutritional and metabolic status of patients undergoing surgical intervention. These studies have highlighted a strong association between severe postoperative complications and the Charlson Comorbidity Index (CCI) > 2 [10,11,12].

According to Suidan et al., CCI was also significantly associated with PFS and overall survival. Median PFS for patients with a CCI value of 0–1, 2–3, and ≥4 was 20.3, 16, and 15.4 months, respectively (*p* = 0.02). Median OS for patients with a CCI value of 0–1, 2–3, and ≥4 was 65.3, 49.9, and 42.3 months, respectively (*p* < 0.001). In a multivariate analysis, the CCI remained a significant prognostic factor for both PFS (*p* = 0.02) and OS (*p* < 0.001) [12].

Cardiovascular disease (left ventricular ejection fraction < 50%, NYHA class III–IV heart failure), poorly controlled diabetes mellitus (HbA1c > 8%), and complex polypharmacy unrelated to oncologic management (more than five medications taken daily) have been identified as factors that negatively impact patient outcomes, being associated with an increased incidence of postoperative complications [7].

Additionally, hypokalemia (<3.7 mmol/L), low INR values (≤0.9), and hyperbilirubinemia have also been reported in some studies as predictors of severe postoperative complications [7,8,9,12].

According to Guelhan Inci et al., patients with a BMI > 25–30 kg/m^2^ are eight times more likely to develop postoperative complications, while those with a BMI > 30 kg/m^2^ have a ninefold increased risk compared to patients with normal body weight [7,12]. These factors, associated with the criteria presented in Table 1, alone or in combination, are highly predictive factors for severe postoperative complications (IIIb-IV) according to the Clavien–Dindo classification.

While frailty is strongly linked to poor outcomes, there is a lack of standardized, practical, and time-efficient tools for assessment in surgical settings. More research is needed to develop and validate tools that can accurately identify frailty in diverse populations and guide perioperative care. In essence, while frailty is increasingly recognized as a crucial factor in surgical outcomes, there are significant gaps in our understanding of how to manage frail patients throughout the perioperative period. Further research is needed to develop better tools for frailty assessment, refine perioperative protocols, and personalize care for this vulnerable population.

### 4.2. Disease-Related Factors—Evaluation of Surgical Complexity and Technical Feasibility

Assessment of operability in patients with ovarian carcinoma is primarily based on the distribution, location, and size of peritoneal carcinomatosis lesions. To evaluate these parameters, several classification systems are employed, the most commonly used being the Fagotti score for laparoscopic staging and the Sugarbaker Peritoneal Cancer Index (PCI) for open surgery [13].

P.J. Fagan et al., in a prospective study published in 2023, reported a single-center experience involving 100 patients with ovarian cancer, analyzing the relationship between PCI scores and the feasibility of achieving R0 resection of carcinomatosis lesions. Cytoreduction was achieved in patients with a mean PCI score of 9, whereas the most accurate predictive factor for incomplete cytoreduction was a mean PCI score of 20. Additionally, major postoperative complications were observed in 15% of cases with a PCI > 20 [13,14].

The prognostic performance of PCI in terms of survival and postoperative complications suggests that patients with PCI > 20 should be referred to NACT and IDS given the increased risk of complications. There is a statistically significant correlation between OS and PCI. An increased PCI is associated with reduced survival.

The ESGO guidelines recommend, for FIGO stage III–IV ovarian cancer, that cytoreductive surgery should be contraindicated in the presence of the factors listed in Table 2 [14,15].

Primary cytoreductive surgery is recommended in all cases where complete resection (R0) can be achieved with an acceptable risk of postoperative complications [13,14].

Surgical management of epithelial ovarian cancer has become increasingly extensive, involving multiorgan resections, which lead to prolonged recovery and hospitalization, as well as higher rates of severe postoperative complications. All these factors contribute to delaying the initiation of chemotherapy beyond 28 days [7,8,15], with a negative impact on survival, particularly in patients with residual disease.

Hofstetter et al. [15], in a prospective study including 191 patients with FIGO stage III and IV disease, demonstrated that a delay in initiating chemotherapy of more than 28 days after surgery adversely affects survival, especially in patients with residual disease (resection < 1 cm). A delay exceeding 42 days results in a 13% reduction in overall survival [15].

A meta-analysis published in 2022 by Kengskul et al. and including over 15,000 patients with ovarian cancer [8,9] identified the following predictive factors for severe postoperative complications within the first 30 days and prolonged convalescence resulting in delayed initiation of chemotherapy: advanced age, FIGO stage IV disease, hypoalbuminemia, and ECOG performance status [11,12].

Although optimal upfront cytoreduction is feasible and has a positive impact on survival, careful preoperative assessment considering these risk factors may reduce morbidity and mortality in patients with impaired general condition and advanced tumor stage.

Given the nonspecific manifestations of ovarian cancer, a significant proportion of patients are diagnosed through imaging performed during the evaluation of nonspecific symptoms, and radiological details of tumor lesions significantly influence therapeutic decision making.

Computed tomography (CT) is the most commonly used investigation method for the detection and staging of ovarian cancer. Additionally, MRI and PET-CT are employed in specialized centers to assess advanced disease cases [16].

R.S. Suidan et al., in a multicenter study conducted over 11 years and including 350 patients, identified 11 clinical and radiologic factors significantly associated with residual postoperative disease > 1 cm, demonstrating a direct correlation between the cumulative score of these factors and the likelihood of incomplete cytoreduction (Table 3) [12,13].

This score includes three clinical and eight radiological criteria, based on which a predictive score for residual disease was obtained. Thus, for patients with values between 0–2, a percentage of 45% of cases with residual disease was recorded, and this percentage increasing to 68% for a value between 3–5 and to 87% for a score with values between 6–8. In the case of patients with a score of 9, residual disease was present in 96% of cases. Although the study includes a large number of patients and is multicenter, it has limitations highlighted by its authors themselves. The evolution of imaging investigations and interpretations obtained and the change in surgical performance over time may alter the predictive value of the score.

In a prospective comparative study evaluating the specificity and sensitivity of computed tomography (CT) versus MRI in staging accuracy and preoperative assessment of sites associated with incomplete cytoreduction, MRI demonstrated higher sensitivity for detecting distant metastases and peritoneal lesions involving the stomach, small intestine, colon, and celiac trunk. MRI showed an accuracy of 95.7% in predicting unresectable disease compared to 71.3% for CT [16,17].

Positron emission tomography (PET-CT) is not recommended for the initial differential diagnosis of ovarian cancer, as its sensitivity in distinguishing borderline lesions from malignant neoplasms is limited. Additionally, endometriotic lesions and hydrosalpinx can produce false-positive results [18].

However, PET-CT is highly useful for detecting nodal metastatic lesions and peritoneal deposits, although miliary disease may be missed. It is particularly valuable in identifying recurrent disease. A recent study confirmed that the highest specificity of PET-CT lies in its detection of extraperitoneal metastases [17,18].

### 4.3. Laparoscopic Assessment

While imaging evaluation may accurately determine resectability in cases of distant metastases, in the peritoneal cavity, specific laparoscopic criteria have been developed and validated to avoid unnecessary laparotomy and to assess the feasibility of achieving complete resection of all visible peritoneal disease (R0).

The most well-known scoring system is that described by Fagotti et al. [19], first published in 2006. This system includes seven parameters assessing the distribution and extent of disease within the peritoneal cavity, assigning a score to each factor based on the characteristics of the lesions. The evaluated parameters are as follows:

1. Peritoneal carcinomatosis lesions: Localized lesions in the paracolic gutters or pelvic peritoneum are considered resecable and scored as 0, while bulky, unresectable lesions receive a score of 2.
2. Diaphragmatic involvement, characterized by multiple disseminated or confluent nodules.
3. Mesenteric involvement.
4. Omental involvement.
5. Bowel involvement.
6. Gastric infiltration.
7. Hepatic metastases.

These parameters provide an indirect estimate of tumor burden, and two points were assigned to each parameter. A score ≥ 8 predicted residual disease with an overall accuracy of 75%, a positive predictive value of 100%, and a negative predictive value of 70%. In 2008, Fagotti et al. validated the performance of the model for estimating optimal cytoreduction (residual disease ≤ 1 cm) in a larger prospective study of advanced ovarian cancer. Although it is directly correlated with PFS and OS, the Fagotti score should not be used as the sole factor in guiding the therapeutic course. Other factors related to the patient’s condition or imaging criteria should also be considered in the decision-making process. Laparoscopic visualization may underestimate the extension of lesions < 1 cm, as well as deep infiltrative tumors [19,20].

In 2018, Hansen et al. published a prospective study comparing the laparoscopic score with intraoperative findings during laparotomy. This study validated the Fagotti score and emphasized its predictive value for achieving R0 resection [20].

### 4.4. Molecular Testing and Surgery

The selection criteria for patients undergoing primary surgical intervention also vary according to histological subtypes and genetic characteristics.

In the case of low-grade serous ovarian carcinoma (LGSOC), neoadjuvant chemotherapy is less effective compared to the response observed in high-grade serous ovarian carcinoma (HGSOC), and cytoreduction with residual tumor < 1 cm is preferable [21,22].

LGSOC accounts for approximately 2% of all epithelial ovarian cancers. While initially classified together with HGSOC, more recent evidence supports its classification as a distinct clinical entity. The majority of LGSOC cases (approximately 60%) are associated with serous borderline tumors, which are considered precursor lesions. Most tumors have ovarian or peritoneal origin, with the latter being associated with a more favorable prognosis. Somatic alterations in the MAPK pathway have been associated with better outcomes, as have advanced age, normal body weight, and non-smoking status [22].

Surgical resection remains the standard of care for LGSOC, adhering to the same principles as in HGSOC, with complete cytoreduction being the most important prognostic factor. In an ancillary analysis of GOG 182, residual postoperative disease was identified as a factor strongly associated with survival (33.2 vs. 14.1 months), favoring patients with microscopic versus macroscopic residual disease. Although therapeutic response to chemotherapy is limited, adjuvant chemotherapy is frequently administered postoperatively. Over 70% of patients with advanced-stage LGSOC experience recurrence. Given the resistance to systemic therapies, surgery remains the first-line approach whenever feasible, often in combination with hormonal therapy or enrollment in clinical trials [22,23].

Current guidelines recommend primary cytoreductive surgery in LGSOC, even in cases with a low likelihood of achieving R0 resection. Similarly, secondary cytoreduction should be considered in recurrent disease, as it significantly impacts disease-free survival (DFS: 60.3 vs. 10.7 months favoring optimal cytoreduction) and overall survival (93.6 vs. 45.8 months favoring surgery) [21].

Selection criteria for surgical intervention in recurrent disease are not yet clearly defined, as most recommendations are extrapolated from studies on HGSOC. Several meta-analyses have demonstrated superior survival outcomes in patients receiving surgery as first-line therapy compared to those treated initially with systemic therapy alone [21,23].

In light of these considerations, surgery is recommended in LGSOC both for newly diagnosed cases and for recurrences, even when complete cytoreduction cannot always be achieved [21,23].

Hyman et al. retrospectively analyzed 367 patients who underwent primary surgery for FIGO stage III–IV HGSOC, of whom 120 had BRCA1/2 mutations. In this cohort, the presence or absence of residual disease and BRCA status were predictive of survival outcomes. Data analysis showed that both age and BRCA status were associated with achieving optimal cytoreduction (initially defined as residual tumor < 1 cm). However, BRCA1/2 mutation status did not independently predict optimal surgical outcome in multivariate analysis, suggesting that improved resection rates in BRCA-mutated cancers were largely attributable to the greater surgical radicality employed in younger patients [24,25,26].

Subsequent studies sought to further analyze the resectability of BRCA1/2-mutated ovarian cancers, this time focusing on the rate of complete macroscopic resection. In 2021, Kim et al. examined a cohort of 303 HGSOC cases, including 120 with BRCA1/2 mutations [27]. Initial clinical presentation, CA-125 levels, surgical complexity scores, and perioperative parameters (hospital stay duration and complication rates) were similar between BRCA-mutated and non-mutated patients. However, a higher rate of complete resection was observed in the BRCA-mutated group (72% vs. 48%). Multivariate analysis confirmed that BRCA1/2 mutation status appeared to be an independent predictor of the likelihood of achieving complete macroscopic resection, irrespective of patient age [26,27].

More recently, Glajzer et al. (2023) confirmed in a cohort of 190 advanced ovarian cancer cases that resectability was significantly improved in BRCA1/2-mutated tumors both in primary and secondary cytoreductive surgeries, despite more extensive abdominal disease distribution [28].

In conclusion, retrospective cohort studies suggest that BRCA1/2-mutated ovarian cancers often present with a substantial disease burden at diagnosis; however, peritoneal lesions tend to exhibit less infiltrative architecture, potentially facilitating easier surgical excision. These observations may explain higher rates of complete resection despite comparable or greater disease extent relative to sporadic tumors [25,26,28].

Therefore, the selection of patients who, based on initial clinical and imaging evaluation, are classified as having a low likelihood of achieving complete cytoreduction, should also consider the histological and molecular characteristics of the tumor.

Currently, biomarkers, such as BRCA gene mutations or homologous recombination deficiency (HRD), are useful for estimating the benefit of PARPi. However, these biomarkers have failed to identify the subgroup of patients who do not respond to PARPi therapy. In addition to the issues related to PARPi and biomarkers, there are other areas of controversy, both in the first line and in relapse, that currently make the management of advanced ovarian cancer more difficult and complex. Our understanding of the genetics and mutations in ovarian cancer is constantly increasing. There is growing recognition that different histological subtypes should be treated as separate entities.

## 5. Discussion

Patients with ovarian cancer represent a population that continues to age, often presenting with compromised nutritional status and physical condition, factors that can impair their ability to tolerate major surgical interventions. Although chronological age alone cannot be considered an independent risk factor for difficult postoperative recovery [29,30], it contributes to the determination of a patient’s frailty score and must be regarded as a factor potentially affecting the capacity to maintain homeostasis after physiological stress [29]. Additionally, older age appears to be associated with poorer survival outcomes [30].

Generally, patient frailty is defined either by the physical phenotype model described by Fried et al. or by the deficit accumulation model, known as the Frailty Index (FI), which quantifies the cumulative burden of deficits by assessing disease status, symptoms, clinical signs, and disabilities in daily living [29,30,31,32,33].

Alongside a limited number of retrospective studies demonstrating the negative impact of frailty on surgical outcomes and survival [31,32,33], prospective data from a recent evaluation of the association between FI, postoperative complications, and overall survival in 144 ovarian cancer patients conducted by Inci et al. [11,29,30] showed that patients with FI > 0.26 (33% of cases) had a fivefold increased risk of developing severe postoperative complications compared to the rest of the cohort. Furthermore, in addition to a residual tumor > 1 cm, an FI > 0.15 was associated with worse overall survival [11,29].

The current literature increasingly recognizes that frailty assessment has already become a key determinant in the decision-making process for therapeutic strategy, influencing outcomes both in major surgery and in the administration of systemic oncologic therapies or enrollment in clinical trials [30].

In addition to identifying frail patients, Narasimhulu et al. proposed an evidence-based algorithm aimed at providing a reproducible and standardized framework for treatment selection to reduce morbidity and mortality associated with debulking surgery. In their study, the risk of adverse postoperative events was significantly increased if at least one of the following parameters was present: albumin < 3.5 g/dL and age ≥ 80 years. Additionally, age between 75–79 years significantly increased the risk of adverse postoperative events when combined with at least one of the following factors: ECOG performance status > 1, FIGO stage IV disease, or the need for complex surgical procedures. In the analyzed cohort of 334 patients with advanced ovarian cancer who underwent either primary debulking surgery (PDS) or neoadjuvant chemotherapy followed by interval debulking surgery (NACT/IDS), according to the selection strategy described, 70% were scheduled for primary surgery, achieving comparable morbidity, mortality, and complete resection rates relative to the IDS group. However, overall survival was better in the cohort selected for PDS [34].

When preoperative assessment of patient frailty and tumor burden is completed, the treatment approach may consist of either PDS or NACT followed by IDS. Generally, when patient condition and tumor distribution allow this, primary cytoreductive surgery followed by platinum-based chemotherapy remains the standard treatment for advanced ovarian cancer [35], as it has been shown to improve survival compared with NACT-IDS [29,35]. For this reason, NACT is considered primarily for patients unlikely to achieve complete cytoreduction-no gross residual disease (NGR) or those who are poor surgical candidates [35], for whom image-guided biopsy represents a valid alternative for obtaining histological confirmation [36].

It is important to note that, when available preoperatively, tumor histotype can play a crucial role in treatment selection. Certain histological subtypes of ovarian cancer are less responsive to chemotherapy, such that primary surgical resection may remain a reasonable option in selected cases despite an unfavorable tumor burden [26,27,28]. In low-grade serous ovarian carcinoma, the survival benefit of NACT appears to be less favorable compared to high-grade serous carcinoma. In these cases, extensive cytoreductive surgery represents the best option to provide a survival advantage, even when tumor burden is substantial. Consequently, surgery in these settings often entails higher surgical complexity scores and more extensive procedures, which in some cases can lead to increased perioperative morbidity [21,22,23]. Similar considerations apply to mucinous tumors, as these patients exhibit poor response to chemotherapy and significantly worse survival compared with other histological subtypes [30].

Given these aspects, the current clinical practice guidelines of the American Society of Clinical Oncology (ASCO) and the Society of Gynecologic Oncology (SGO) state that, when there is a high probability of achieving no gross residual disease or residual tumor < 1 cm with acceptable morbidity, PDS should be the preferred treatment option [27]. Despite this, the superior outcomes reported in patients treated with PDS compared to NACT remain a subject of ongoing debate [26,27,29].

The publication of the results of the ENGOT ov33/AGO-OVAR op.7 trials, preliminarily presented at ASCO 2025, could answer these questions.

In this context, several phase III studies have investigated whether NACT-IDS is as effective and safe as PDS followed by platinum-based chemotherapy in this population [28,36]. In the EORTC-55971 trial, 670 women with FIGO stage IIIC/IV ovarian cancer were randomized to receive NACT-IDS versus PDS, demonstrating similar median survival between the two groups (29 vs. 30 months, respectively) but with lower surgical morbidity in the NACT cohort [37,38]. Comparable survival outcomes were observed in the CHORUS trial [36], which randomized 550 patients with advanced ovarian cancer to the two treatment arms, again demonstrating similar survival (22.8 months for PDS vs. 24.5 months for NACT-IDS). Subsequently, a combined individual patient data analysis from EORTC 55,971 [38] and CHORUS [36] highlighted improved survival among FIGO stage IV patients treated with NACT-IDS, with a median overall survival of 24.3 months for NACT-IDS compared to 21.2 months for PDS (*p* = 0.48) and median progression-free survival of 10.6 versus 9.7 months (*p* = 0.049) [37,38,39].

Marchetti et al., in a randomized study of 171 patients, demonstrated no differences in patient-reported quality of life at 12 months between treatment strategies (PDS or NACT-IDS), supporting the feasibility of interval surgery as an alternative for patients not eligible for PDS [40].

In a systematic review published in 2023, which included 14 studies totaling 1813 patients, Della Corte et al. showed that in patients with NACT followed by surgery, the combination of HIPEC with IDS brought a significant benefit in progression-free survival (PFS) and OS (overall survival), compared to patients who underwent surgery alone. Thus, between the two groups, a PFS of 5.86 months was recorded in favor of the combination of HIPEC compared to 4.68 months for surgery alone and OS of 14.27 compared to 11.8 months, all of this with an acceptable morbidity [41].

In preoperative evaluation of EOC, assessing PARP (poly ADP–ribose polymerase) status and HRD (homologous recombination deficiency) status has become increasingly important for guiding treatment strategies and predicting outcomes. Knowing HRD status preoperatively helps in planning targeted therapies, especially in cases where upfront surgery might be followed by maintenance therapy with PARP inhibitors and also when patients may benefit from neoadjuvant chemotherapy combined with PARP inhibitors, potentially impacting surgical decisions.

PARPi represents a major change in the treatment of high-grade EOC. All studies have shown improvements in survival and progression-free survival (PFS) with maintenance treatment with PARPi. Current debates focus on their use and incorporation into different phases of treatment [42,43].

Given the complexity of the factors taken into account in the triage of patients with EOC, the decision and the therapeutic pathway are the subject of the multidisciplinary oncologic board. The development of AI and its testing in the selection of oncological patients has had promising results, but further evaluations on large groups of patients are needed to validate this technology in selecting the therapeutic pathway of patients with EOC [44].

## 6. Conclusions

In conclusion, within a context where treatment paradigms are progressively evolving from a binary PDS-IDS approach toward a more individualized therapeutic strategy, a thorough assessment of patient condition, overall performance status, histological and molecular tumor characteristics, as well as tumor burden, is essential for appropriate treatment planning.

Such evaluations are recommended to be conducted in highly specialized centers with extensive experience in oncologic surgery, in order to maximize outcomes for patients with ovarian cancer, given both the complexity of patient selection factors and the intricacy of surgical procedures.

## Figures and Tables

**Table 1:** Predictive factors for postoperative high morbidity and poor prognostic.

Predictive Factors for Poor Survival	
ECOG	≥3
Left ventricular ejection fraction	<50%
Frailty	>0.26
Hypoalbuminemia	<35.5 g/dL
Residual disease	>1 cm
BMI	>25–30 Kg/m^2^
CCI (Charlson Comorbidity Index)	>2

**Table 2:** Criteria against abdominal debulking, according to ESGO guidelines (2017) [15].

Diffuse infiltration of the root of SB (small bowel) mesentery
Diffuse carcinomatosis of the SB involving large parts so the resection will lead to short bowel syndrome
Diffuse involvement/deep infiltration of the stomach, duodenum, and head or middle part of the pancreas
Involvement of truncus celiacs with the hepatic artery and left gastric artery
Central or multisegmental parenchymal liver metastases
Multiple parenchymal lung metastases (if possible, histologically proven)

**Table 3:** Clinical and radiological predictive criteria of gross residual disease (R.S. SUIDAN et al.) [12].

Criteria	Predictive Score
AGE ≥ 60	1
CA-125 ≥ 600 u/ML	1
ASA ≥ 3 Lesion in splenic hilum/ligaments	1
Gastrohepatic ligament/portal lesion	1
Paraaortic lymph nodes above the renal hilum (including supradiaphragmatic)	1
Diffuse small bowel thickening	1
Abdominal ascites (moderate–severe)	2
Gallbladder fossa/intersegmental fissure lesion	2
Lesser sac lesion > 1 cm	2
SMA lesion (root)	4
SMA (superior mesenteric artery)	ASA (American Society of Anesthesiologists)
